# Iron Acquisition and Siderophore Release by Carbapenem-Resistant Sequence Type 258 *Klebsiella pneumoniae*

**DOI:** 10.1128/mSphere.00125-18

**Published:** 2018-04-18

**Authors:** Victoria I. Holden, Meredith S. Wright, Sébastien Houle, Abigail Collingwood, Charles M. Dozois, Mark D. Adams, Michael A. Bachman

**Affiliations:** a Department of Microbiology and Immunology, University of Michigan, Ann Arbor, Michigan, USA; b J. Craig Venter Institute, La Jolla, California, USA; c Institut National de Recherche Scientifique, Institut Armand Frappier, Laval, Canada; d Department of Pathology, University of Michigan, Ann Arbor, Michigan, USA; JMI Laboratories

**Keywords:** CRE, *Klebsiella pneumoniae*, ST258, carbapenems, *entS*, enterobactin, hemin

## Abstract

Klebsiella pneumoniae is rapidly acquiring resistance to all known antibiotics, including carbapenems. Multilocus sequence type ST258 (sequence type 258), carrying a gene encoding the K. pneumoniae carbapenemase (*bla*_KPC_) on a transmissible plasmid, is the most prevalent carbapenem-resistant *Enterobacteriaceae* (CRE) in the United States and has disseminated worldwide. Previously, whole-genome sequencing identified core genome single nucleotide variants that divide ST258 into two distinct clades, ST258a and ST258b. Furthermore, a subset of ST258b strains have a 347-base deletion within the enterobactin (Ent) exporter gene *entS*. Despite the predicted inability of these strains to secrete the siderophore Ent, this clade is prevalent among clinical isolates, indicating that a full-length *entS* gene is not necessary for infection. To compare the transcriptional responses of ST258 subtypes to iron limitation, we performed transcriptome sequencing (RNA-Seq) in minimal medium alone or supplemented with iron or human serum and measured gene expression patterns. Iron limitation induced differential expression of distinct iron acquisition pathways when comparing ST258a and ST258b strains, including the upregulation of the hemin transport operon in *entS* partial deletion isolates. To measure how K. pneumoniae strains vary in iron chelation and siderophore production, we performed *in vitro* chrome azurol S (CAS) and Arnow assays as well as mass spectrometry. We determined that both ST258a and ST258b strains grow under iron-depleted conditions, can utilize hemin for growth, and secrete Ent, despite the partial *entS* deletion in a subset of ST258b strains. All carbapenem-resistant (CR) K. pneumoniae strains tested were susceptible to growth inhibition by the Ent-sequestering innate immune protein lipocalin 2.

**IMPORTANCE** Carbapenem-resistant *Enterobacteriaceae*, including K. pneumoniae, are a major health care concern worldwide because they cause a wide range of infection and are resistant to all or nearly all antibiotics. To cause infection, these bacteria must acquire iron, and a major mechanism of acquiring iron is by secreting a molecule called enterobactin that strips iron from host proteins. However, a subset of carbapenem-resistant K. pneumoniae strains that lack a portion of the *entS* gene that is required for enterobactin secretion was recently discovered. To understand how these mutant strains obtain iron, we studied their transcriptional responses, bacterial growth, and enterobactin secretion under iron-limited conditions. We found that strains both with mutated and intact *entS* genes grow under iron-limiting conditions, secrete enterobactin, and utilize an alternate iron source, hemin, for growth. Our data indicate that carbapenem-resistant K. pneumoniae can use varied methods for iron uptake during infection.

## Abstract (precis)

Carbapenem-resistant *Enterobacteriaceae*, including K. pneumoniae, are a major health care concern worldwide because they cause a wide range of infection and are resistant to all or nearly all antibiotics. To cause infection, these bacteria must acquire iron, and a major mechanism of acquiring iron is by secreting a molecule called enterobactin that strips iron from host proteins. However, a subset of carbapenem-resistant K. pneumoniae strains that lack a portion of the *entS* gene that is required for enterobactin secretion was recently discovered. To understand how these mutant strains obtain iron, we studied their transcriptional responses, bacterial growth, and enterobactin secretion under iron-limited conditions. We found that strains both with mutated and intact *entS* genes grow under iron-limiting conditions, secrete enterobactin, and utilize an alternate iron source, hemin, for growth. Our data indicate that carbapenem-resistant K. pneumoniae can use varied methods for iron uptake during infection.

## INTRODUCTION

Carbapenem-resistant *Enterobacteriaceae* (CRE) are resistant to all or nearly all antibiotics, are increasingly present worldwide, and are therefore designated an urgent public health concern (1). Infection with carbapenem-resistant (CR) Klebsiella pneumoniae is of particular concern due to its high mortality rate of approximately 41 to 50% for bloodstream infections (1–3). *Klebsiella* species cause a variety of infections, including pneumonia, urinary tract infection, and bacteremia, and represent the third most common cause of health care-acquired infection, further contributing to their relevance to health care settings (4). Despite epidemiological studies documenting the spread of infection and patient risk factors associated with infection, the bacterial pathogenesis of CR K. pneumoniae is currently not well understood (5–7).

To measure the genomic diversity of CR K. pneumoniae, a cohort of isolates was collected from hospitals within the midwestern United States for whole-genome sequencing (8, 9). Strains isolated from infection and colonization were primarily the multilocus sequence type (MLST) ST258 (sequence type 258), but molecular genotyping based on core genome single nucleotide variants separated them into two distinct clades, ST258a and ST258b (8, 9). ST258a isolates were significantly associated with the carbapenemase *bla*_KPC-2_, whereas ST258b isolates were associated with the carbapenamase *bla*_KPC-3_ (8). Significant epidemiological differences were present as well. Infection with ST258a isolates was more likely to result in an increased length of hospital stay than ST258b infection, and ST258b infection was more commonly acquired from a skilled nursing facility, whereas ST258a infections were more commonly community acquired (10). These differences in presentation and outcome suggested functional differences between these clades during infection. Indeed, whole-genome comparisons uncovered numerous polymorphisms within the capsular polysaccharide locus and extending into the *mdtABC* locus, as well as differences in plasmid contents (8, 9). Intriguingly, a 347-bp deletion in the enterobactin (Ent) siderophore exporter gene *entS* (*entS*Δ_347_) was found in a subset of ST258b isolates that was absent in ST258a isolates (9). The *entS* nucleotide sequence of ST258a isolates contains a 10-nucleotide repeat, and the deletion in ST258b isolates begins after the first instance and ends immediately after the second repeat (nucleotides 324 to 670; see Fig. S1A in the supplemental material). This mutated allele (*entS*Δ_347_) contains a frameshift, altering the amino acid sequence beginning at position 109 and resulting in a premature stop codon truncating the protein at position 136 compared to the full-length 413-amino-acid sequence (Fig. S1B).

10.1128/mSphere.00125-18.1 FIG S1  Alignments of *entS* and *entS*Δ_347_ alleles and encoded proteins. Nucleotide sequence alignment (A) demonstrates a 347-bp deletion in the region between 10-nucleotide repeats (bold), creating a frameshift and stop codon (bold italics) that results in a protein sequence (B) that is altered (underlined) and truncated in a subset of ST258b isolates. Accession numbers: AEW60142.1 for ST258a and GCF_000709205.1 for ST258b (nucleotides 4498971 to 4499865). Download FIG S1, PDF file, 0.05 MB. Copyright © 2018 Holden et al. 2018 Holden et al. This content is distributed under the terms of the Creative Commons Attribution 4.0 International license.

Ent is the prototypic catecholate siderophore and is secreted by Gram-negative bacteria, including K. pneumoniae, to acquire iron (11). Siderophores are major virulence factors in K. pneumoniae, and Ent has the highest known affinity for iron of any molecule (11). To counter the iron-sequestering effects of Ent, host cells secrete lipocalin 2 (Lcn2) that specifically binds to and sequesters Ent. Because *entS* encodes the only known Ent exporter, a deletion in *entS* is predicted to impair the ability of bacteria to secrete Ent for iron acquisition (12). Because K. pneumoniae ST258 isolates do not carry genes encoding additional siderophores, the inability to secrete Ent could be deleterious. In Escherichia coli, *entS*-null mutants release Ent breakdown products that have lower affinity for iron than that of intact Ent. The Ent esterase Fes cleaves cyclic Ent into monomers, dimers, and trimers of 2,3-dihydroxybenzoylserine (DHBS) (13, 14). Whereas the affinity of the linearized trimer of DHBS is similar to that of Ent, the iron affinities of DHBS monomers and dimers are less than that of Ent (15). Despite their lower affinities, some of these breakdown products can act as siderophores (13, 14). In extraintestinal pathogenic E. coli, an *entS* mutant is significantly attenuated in a systemic infection model (12). However, in K. pneumoniae, conservation of the *entS*Δ_347_ allele within a subset of the ST258b clade suggests that compensatory iron acquisition or alternative Ent transport mechanisms may be present. A decrease in the export of Ent in favor of release of linear DHBS molecules and use of alternate compensatory iron acquisition pathways could be a strategy for some K. pneumoniae strains to evade the bacteriostatic effects of Lcn2 during infection.

To understand iron acquisition by CR K. pneumoniae, we examined the transcriptional and phenotypic responses of ST258 isolates to iron limitation induced by incubation in minimal medium and human serum. To study gene transcription in response to iron-replete and iron-depleted conditions, we performed transcriptome sequencing (RNA-Seq) to identify differential gene expression based on iron status and *entS* deletion. Additionally, we performed *in vitro* assays to characterize the ability of ST258a and ST258b strains to grow in iron-limiting medium and to secrete iron-chelating molecules. We characterized the secretion of Ent by these strains using mass spectrometry. These results serve to increase the general understanding of CR K. pneumoniae iron acquisition and identify distinct iron starvation responses by ST258a and ST258b CR K. pneumoniae.

## RESULTS

### RNA-Seq analysis identifies differential gene expression in response to iron-depleted media.

**FIG 1:**
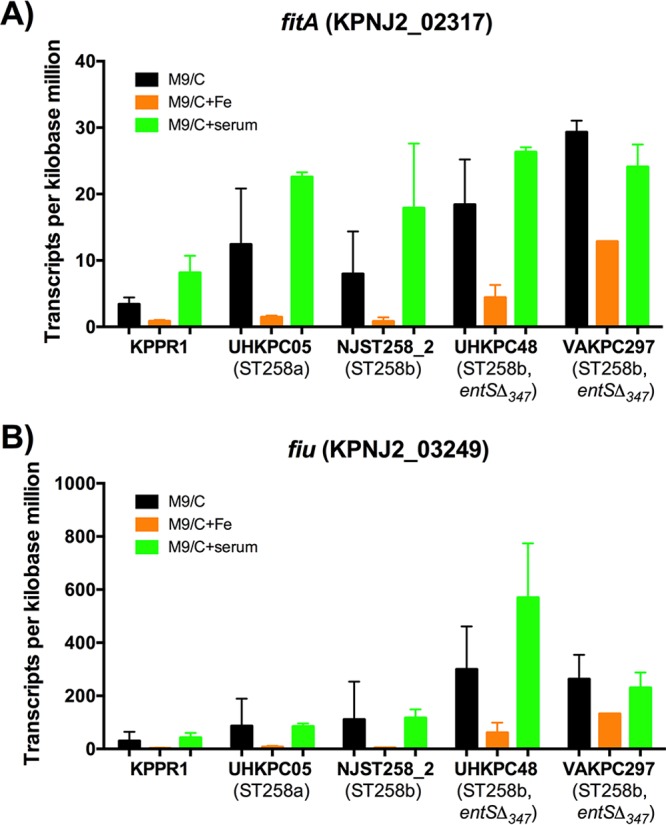
Outer membrane receptor genes *fitA* and *fiu* are upregulated in ST258 isolates during iron limitation. Transcripts per kilobase million values for each isolate were analyzed. (A and B) The *fitA* and *fiu* genes were significantly upregulated by ST258 strains in M9/Chelex (M9/C) compared to M9/Chelex+Fe (M9/C+Fe). Data were analyzed using the DESeq2 package in R.

**FIG 2:**
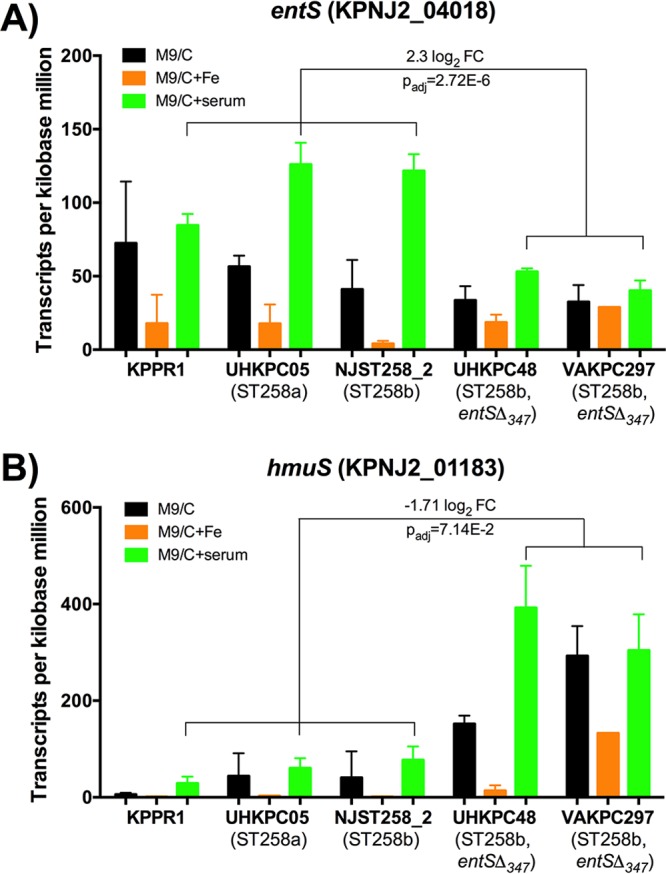
ST258b *entS*Δ_347_ strains express greater *hmuS* than other ST258 strains during iron limitation. (A and B) Transcripts per kilobase million values for each isolate were analyzed for the Ent exporter *entS* and hemin transporter *hmuS* in M9/Chelex (M9/C) and M9/Chelex+serum (M9/C+serum). Data were analyzed using the DESeq2 package in R. FC, fold change; P_adj_, adjusted *P* value.

The representative strains of *K*. *pneumoniae* utilized in this study are shown in Table 1. To examine the effect of the *entS*Δ_347_ allele on the transcriptional response to iron-limited media, RNA-Seq was performed on the following strains: KPPR1, a wild-type (WT) strain secreting the siderophores Ent, yersiniabactin (Ybt), and salmochelin (Sal); UHKPC05, an ST258a strain with a full-length *entS* gene; NJST258_2, an ST258b strain with a full-length *entS* gene; UHKPC48 and VAKPC297, ST258b strains with the *entS*Δ_347_ allele. Samples were collected for RNA-Seq 1 h after subculture into iron-limited minimal medium alone (M9/C) or medium supplemented with iron (M9/C+Fe) or 5% human serum (M9/C+serum; see Fig. S2 in the supplemental material). To identify genes among all ST258 isolates that were significantly modulated under iron-depleted conditions (iron-depleted M9 minimal medium [M9/Chelex] versus iron-replete M9 medium [M9/Chelex+Fe]), the DESeq2 package in R was used to analyze count data obtained from RNA-Seq (Table 2). Negative fold changes represent genes that were significantly downregulated in response to iron depletion, whereas positive fold changes represent genes that were significantly upregulated in response to iron depletion. Genes that were significantly downregulated in response to iron-depleted media include the iron storage protein ferritin gene (KPNJ2_01976) and superoxide dismutase *sodB* (KPNJ2_02431) that uses iron as a cofactor. The downregulation of these iron-dependent genes during iron-depleted conditions serves as an internal validation. Genes that were significantly upregulated in response to iron-depleted media include Ent synthesis genes (reference gene loci KPNJ2_04011-04015), an iron-sulfur cluster assembly locus (KPNJ2_02279-02282), a proposed TonB-dependent siderophore receptor *fitA* (Fig. 1A), a catecholate siderophore receptor *fiu* locus (Fig. 1B), and a hemin transport system gene (KPNJ2_01180-01184). To identify transcriptional differences associated with the *entS*Δ_347_ allele, gene expression in *entS*^*+*^ and *entS*Δ_347_ isolates were compared in the physiologically relevant iron-limited condition M9/Chelex+serum. Expression of *entS* was reduced in *entS*Δ_347_ isolates (Fig. 2A, 2.3 log_2_ fold change and adjusted *P* value of 2.72E−6). In contrast, gene expression of the hemin transporter *hmuS* was significantly higher in *entS*Δ_347_ isolates than in *entS*^*+*^ isolates (Fig. 2B, −1.71 log_2_ fold change and adjusted *P* value of 7.14E−2).

10.1128/mSphere.00125-18.2 FIG S2  Bacterial CFU during RNA-Seq experiment. Once bacteria reached mid-log growth phase, an initial aliquot was reserved and plated for CFU. Strains were then subcultured into M9/Chelex, M9/Chelex plus 100 µM Fe_2_SO_4_, and M9/Chelex plus 5% HI serum. After 1 h, aliquots were taken from each condition and plated for CFU. Data presented are means ± standard deviation of two technical replicates of duplicate samples. Download FIG S2, PDF file, 0.1 MB. Copyright © 2018 Holden et al. 2018 Holden et al. This content is distributed under the terms of the Creative Commons Attribution 4.0 International license.

**TABLE 1:** K. pneumoniae strains used in this work

Strain	Relevant characteristic(s)^a^	ST258 clade	*entS*
KPPR1	Ent+ Ybt+ Sal+	−	+
KP7	KPPR1 *entB ybtS*; siderophore-negative	−	+
KP20	KPPR1 *iroA ybtS*; Ent+	−	+
UHKPC05	Ent+	A	+
NJST258_1	Ent+	B	+
NJST258_2	Ent+	B	+
UHKPC48	Ent+	B	Δ_347_
DMC0526	Ent+	B	Δ_347_
VAKPC297	Ent+	B	Δ_347_

a Ent+, Ybt+, or Sal+ indicates that the strain carries the gene encoding the corresponding siderophore (e.g., *ent*).

**TABLE 2:** Gene transcripts that differ significantly in iron-limited medium compared to iron-replete medium among all ST258 isolates^a^

Gene	Predicted annotation	KPRR1			NJST258_2			UHKPC05			UHKPC48			VAKPC297			Log_2_ fold change	Adjusted *P* value
		M9+Fe	M9	Serum	M9+Fe	M9	Serum	M9+Fe	M9	Serum	M9+Fe	M9	Serum	M9+Fe	M9	Serum		
KPNJ2_00325	Ccm1 protein	1.8	7.4	9.9	2.3	8.7	14.4	3.7	19.4	15.1	8.5	21.3	20.0	12.8	27.1	21.8	1.7	7.19E−05
KPNJ2_01180	Hemin transport system ATP-binding protein HmuV	1.0	1.7	17.7	0.4	49.9	58.4	3.6	38.6	43.6	13.1	120.7	251.4	51.5	150.4	157.2	3.1	1.54E−05
KPNJ2_01181	Hemin transport system permease protein HmuU	0.3	0.8	6.3	0.1	15.7	34.0	2.1	18.7	25.0	7.8	72.7	155.1	43.9	106.9	99.0	3.0	5.17E−06
KPNJ2_01182	Hemin-binding periplasmic protein HmuT precursor	0.3	1.3	14.9	0.8	31.5	46.3	1.9	34.1	36.6	11.7	122.8	242.8	73.9	187.0	143.3	3.3	1.51E−06
KPNJ2_01183	Hemin transporter HmuS	1.0	5.7	28.8	1.2	40.5	77.0	2.7	43.7	60.4	13.5	152.0	392.1	132.6	292.7	304.3	3.3	3.13E−08
KPNJ2_01184	TonB-dependent receptor	4.8	11.7	28.9	4.4	37.9	85.9	4.8	44.4	59.1	14.4	142.7	336.4	121.1	304.2	304.4	2.5	2.11E−07
KPNJ2_01262	Ribonucleoside diphosphate reductase subunit beta NrdF2	24.6	42.7	216.8	21.7	144.7	847.5	50.8	273.7	549.0	52.2	152.2	771.2	169.8	240.6	838.3	1.6	1.28E−05
KPNJ2_01263	Ribonucleoside diphosphate reductase alpha chain	4.4	21.0	98.5	14.0	72.0	499.7	24.8	140.9	349.3	27.2	83.5	473.6	104.6	160.2	503.2	1.8	3.68E−08
KPNJ2_01264	NrdI protein	1.1	4.2	29.4	3.9	38.5	198.4	8.6	56.9	145.3	9.1	31.0	198.3	40.8	81.3	228.8	2.1	1.75E−07
KPNJ2_01265	Glutaredoxin	5.0	2.6	20.7	2.5	78.1	503.7	31.4	119.2	249.8	18.6	62.3	428.2	58.8	111.4	408.7	1.7	5.11E−05
KPNJ2_01266	Carboxymuconolactone decarboxylase family protein	4.5	16.1	59.7	2.7	13.6	40.0	6.6	20.6	50.4	10.6	31.3	66.2	30.5	58.2	45.0	1.6	5.11E−05
KPNJ2_01377	Hypothetical protein	12.0	14.4	17.8	1.8	25.3	18.5	15.6	29.6	28.5	14.9	31.1	28.5	0.7	15.2	22.6	1.7	1.59E−04
KPNJ2_01378	Sulfite reductase (NADPH) flavoprotein alpha-component	9.2	45.6	90.6	4.6	104.2	116.7	54.6	198.8	142.5	11.7	242.5	208.7	5.3	131.4	192.6	3.5	9.82E−22
KPNJ2_01379	Lipoprotein	14.5	23.0	27.6	9.1	40.5	25.3	22.7	51.6	27.5	18.0	61.3	45.5	4.8	31.7	42.6	1.6	1.35E−05
KPNJ2_01730	Catecholate siderophore receptor CirA	48.6	639.2	2091.9	34.0	343.9	899.4	74.8	738.0	1097.4	400.5	1363.7	2053.9	893.7	1177.0	1814.5	2.5	1.84E−07
KPNJ2_01976	Ferritin	264.7	36.4	128.5	607.4	85.9	45.2	186.8	31.7	34.4	45.5	23.0	29.1	58.8	44.4	56.4	−2.0	1.14E−06
KPNJ2_02010	Zinc ABC transporter substrate-binding protein	95.2	17.0	44.1	68.9	49.3	62.8	195.2	71.4	75.8	145.3	50.4	58.6	362.0	55.9	85.5	−1.8	1.02E−10
KPNJ2_02086	Ferrioxamine B receptor	13.1	70.4	177.6	12.8	55.9	165.6	14.6	70.2	126.1	33.7	92.1	144.6	127.5	151.8	178.0	1.7	2.05E−08
KPNJ2_02279	Iron-sulfur cluster assembly protein SufA	6.7	14.9	32.0	18.4	152.4	409.2	51.4	302.9	640.8	63.6	233.9	413.9	147.1	447.8	595.9	2.0	6.73E−06
KPNJ2_02280	Cysteine desulfurase	9.3	18.6	30.7	26.7	146.4	435.5	47.0	312.2	660.9	71.0	282.4	434.3	197.0	516.3	566.0	1.9	3.52E−06
KPNJ2_02281	FeS assembly ATPase SufC	11.8	16.7	37.9	25.5	122.5	467.3	48.0	261.9	619.4	82.2	252.6	484.9	195.1	402.1	582.7	1.6	5.04E−04
KPNJ2_02282	FeS assembly protein SufD	7.2	10.2	21.4	11.1	41.7	119.4	18.8	83.2	195.7	25.2	91.9	147.9	60.6	157.2	183.8	1.6	3.07E−04
KPNJ2_02317	TonB-dependent outer membrane ferric coprogen receptor FitA	0.8	3.4	8.1	0.8	8.0	17.9	1.5	12.4	22.6	4.4	18.4	26.3	12.9	29.3	24.1	2.3	4.71E−11
KPNJ2_02319	ABC transporter	4.0	17.0	22.2	3.0	19.8	31.5	7.0	20.7	31.9	9.6	21.8	28.1	24.0	31.8	21.2	1.6	8.98E−06
KPNJ2_02431	*sodB*	401.7	225.5	36.5	503.6	104.9	19.1	426.0	58.8	20.3	136.4	36.5	28.2	48.6	30.3	19.6	−1.8	4.01E−05
KPNJ2_02525	Hypothetical protein	9.9	165.8	495.1	36.8	225.8	531.7	75.8	361.3	406.0	195.3	509.6	657.8	526.6	649.4	600.4	2.1	1.83E−05
KPNJ2_02780	IroE protein	21.7	64.5	115.8	11.6	52.7	115.4	25.2	102.5	103.8	62.4	141.7	206.4	123.7	194.5	169.7	1.5	3.98E−07
KPNJ2_03249	Catecholate siderophore receptor Fiu	2.8	30.1	42.4	4.3	110.8	117.0	7.5	86.2	84.7	60.6	299.8	569.3	131.9	263.2	230.1	3.0	5.70E−07
KPNJ2_03250	Iron uptake factor	7.3	17.3	16.2	5.7	30.7	19.5	2.1	19.7	15.6	10.7	44.3	69.3	20.4	38.6	34.8	2.0	2.97E−04
KPNJ2_03409	Ligand-gated channel protein	41.3	136.6	171.4	23.6	184.4	252.8	82.1	285.3	226.8	194.3	376.1	410.0	234.5	327.5	287.0	1.6	1.06E−02
KPNJ2_04011	Proofreading thioesterase in enterobactin biosynthesis	7.9	68.1	132.2	7.3	70.7	174.7	25.7	101.9	198.5	65.4	157.2	205.8	201.0	151.3	269.3	1.8	4.56E−03
KPNJ2_04012	*entA*	10.9	65.3	130.9	19.5	180.0	203.2	47.0	236.5	221.9	96.2	299.6	293.0	153.6	283.7	290.3	2.1	1.34E−05
KPNJ2_04013	Isochorismatase	13.2	139.7	257.4	24.4	258.8	399.6	90.7	489.9	458.6	196.8	592.8	590.6	319.7	493.9	519.3	2.3	1.18E−05
KPNJ2_04014	2,3-Dihydroxybenzoate-AMP ligase	28.3	156.0	245.9	18.3	256.3	421.6	62.4	337.8	388.9	173.7	499.9	515.2	259.6	459.3	528.8	2.2	2.47E−05
KPNJ2_04015	EntC isochorismate synthase	43.6	261.2	503.6	28.0	294.1	600.4	81.9	512.9	589.1	209.6	643.6	724.5	397.8	683.5	814.7	2.2	1.28E−05
KPNJ2_04017	Ferrienterobactin-binding protein	20.1	79.6	164.6	11.2	59.0	131.2	23.7	116.0	169.1	57.5	141.2	141.5	129.2	159.2	178.9	1.6	2.18E−06
KPNJ2_04018	EntS MFS^b^ transporter	17.8	72.4	84.6	4.1	41.0	121.6	17.7	56.5	126.0	18.5	33.6	53.1	28.9	32.5	40.2	1.6	1.19E−04
KPNJ2_04023	Enterobactin synthase subunit F	10.0	59.4	141.7	8.1	65.0	220.4	22.4	97.0	226.1	53.8	105.3	162.8	105.7	121.2	193.0	1.8	9.69E−04
KPNJ2_04025	Enterochelin esterase	7.4	31.6	44.6	2.1	32.3	94.9	11.0	39.2	100.5	23.0	40.7	74.9	38.9	51.5	88.1	1.8	9.69E−04
KPNJ2_04026	Outer membrane receptor protein	47.1	799.3	1060.6	35.1	495.5	1107.4	112.6	586.0	1118.4	331.8	954.4	1375.3	985.3	1024.8	1396.3	2.4	1.09E−06
KPNJ2_04027	Enterobactin synthase EntD component	2.9	23.4	63.9	3.3	79.9	129.1	16.1	99.3	183.3	44.9	136.4	148.3	101.2	118.4	140.3	2.4	9.85E−13
KPNJ2_04084	TonB-dependent receptor	2.7	29.2	38.3	1.0	23.5	44.9	7.2	58.7	52.4	14.6	65.3	80.3	51.2	67.2	90.3	2.7	8.07E−07
KPNJ2_04085	Hypothetical protein	13.6	127.1	182.8	7.0	53.7	177.6	16.2	158.2	203.8	43.0	216.3	291.0	192.6	259.2	303.4	2.5	2.06E−07
KPNJ2_04207	Ribosomal protein L31	116.7	57.1	34.4	28.7	17.3	29.3	97.7	28.8	48.8	220.6	22.7	35.5	240.5	26.1	41.6	−2.0	1.51E−06
KPNJ2_04728	Hypothetical protein	9.9	69.5	236.0	26.0	81.5	525.5	39.2	260.2	952.2	49.2	272.0	533.2	355.9	681.9	847.1	2.1	9.28E−14
KPNJ2_05307	Zinc-cadmium-binding protein	97.2	25.0	17.4	25.0	18.4	15.5	40.3	26.9	22.1	88.6	17.8	23.1	118.2	15.2	19.0	−1.7	8.89E−06

a Gene transcripts that differ significantly (log_2_ fold change of >1.5; adjusted *P* value of <0.05) in iron-limited medium (M9/Chelex) compared to iron-replete medium (M9/Chelex+Fe). Data are represented as transcripts per kilobase million. Log_2_ fold change and adjusted *P* value identify genes significantly up- or downregulated by all ST258 genes in response to iron depletion.

b MFS, major facilitator superfamily.

### Growth of K. pneumoniae ST258 strains in iron-replete and iron-depleted conditions.

**FIG 3:**
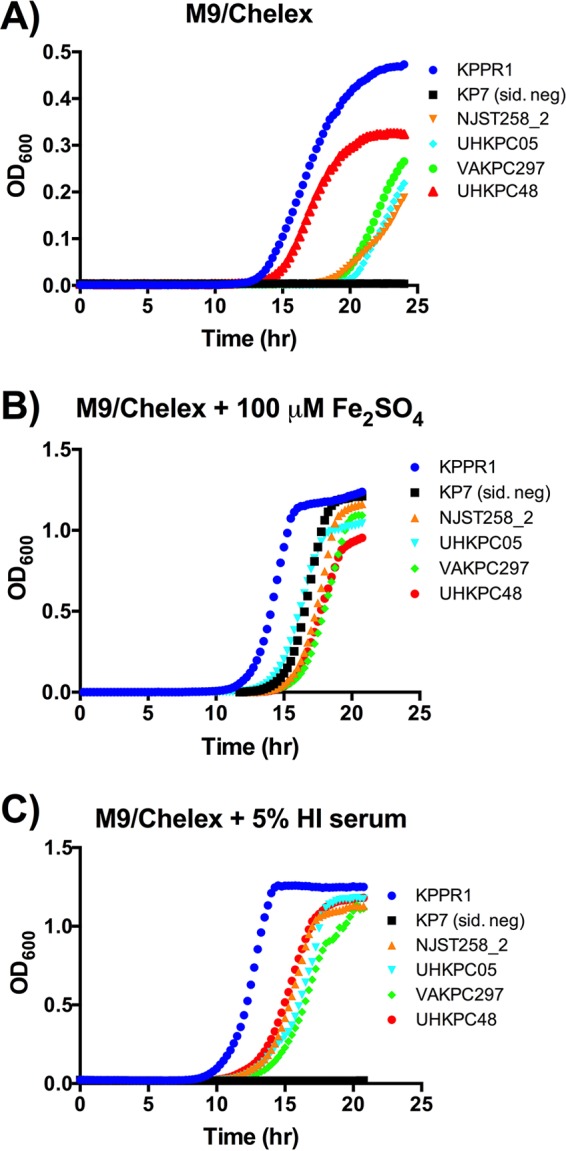
Isolates from both ST258 clades replicate in iron-limited media. (A to C) Strains were cultured overnight in LB, then subcultured at 1:100,000 into a 96-well plate for M9/Chelex, M9/Chelex plus 100 µM Fe_2_SO_4_, and M9/Chelex plus 5% heat-inactivated (HI) serum. Strains were cultured overnight, and OD_600_ was read at 15-min intervals. Data are representative of two or three individual replicates. Strain KP7 is siderophore negative (sid. neg).

To determine the ability of ST258 strains to grow under iron limitation, cultures were extensively diluted (1:100,000) into iron-depleted M9 minimal medium (M9/Chelex), and growth curves were constructed. Under these conditions, an E. coli* entS*-null mutant had a small but consistent growth defect (Fig. S3), suggesting that a functional defect in ST258 *entS*Δ_347_ mutants may be detected. Because the extreme antibiotic resistance of ST258 eliminates use of many genetic markers, it is technically challenging to create isogenic mutants. Therefore, the KPPR1 strain and an isogenic siderophore-negative mutant KP7 were used as positive and negative controls for siderophore-dependent growth, respectively. KPPR1 had the earliest and maximal growth, whereas KP7 was unable to grow (Fig. 3A). The ST258 strains grew but compared to KPPR1, the UHKPC48 strain had a slight delay and NJST258_2, UHKPC05, and VAKPC297 strains had longer delays. Although strains UHKPC48 and VAKPC297 both express the *entS*Δ_347_ allele, the delay in growth of the VAKPC297 isolate is more dramatic than the growth delay of UHKPC48. With the addition of 100 µM Fe_2_SO_4_, the growth of KP7 was rescued, and the growth of NJST258_2, UHKPC05, and VAKPC297 improved (Fig. 3B).

10.1128/mSphere.00125-18.3 FIG S3  An E. coli* entS*-null mutant has a growth defect in M9/Chelex. Strains were cultured overnight in LB, subcultured to a concentration of ~1e5 CFU/ml in M9/Chelex, cultured overnight, and OD_600_ was read at 15-min intervals. Data are the means ± standard deviations for nine replicates from four independent experiments. **, *P* < 0.01 comparing the final OD_600_ by *t* test. Download FIG S3, PDF file, 0.1 MB. Copyright © 2018 Holden et al. 2018 Holden et al. This content is distributed under the terms of the Creative Commons Attribution 4.0 International license.

Within serum, iron is bound by the host protein transferrin, and iron acquisition for growth by WT KPPR1 requires siderophores (16). To examine the ability of the strains to acquire iron in a physiologically relevant iron-limiting condition, growth curves were performed in M9/Chelex supplemented with 5% heat-inactivated serum. Similar to M9, strain KPPR1 had maximal growth and strain KP7 did not grow. However, all ST258 strains grew rapidly in media containing 5% serum as an iron source, indicating that ST258 strains are able to acquire iron from human serum for replication (Fig. 3C). These data document that all ST258 strains, even those with the *entS*Δ_347_ allele, are able to grow in various iron-depleted and iron-replete growth conditions, although they did not grow as well as KPPR1 strain, which secretes multiple siderophores.

### ST258 strains secrete iron-chelating molecules and catechols.

**FIG 4:**
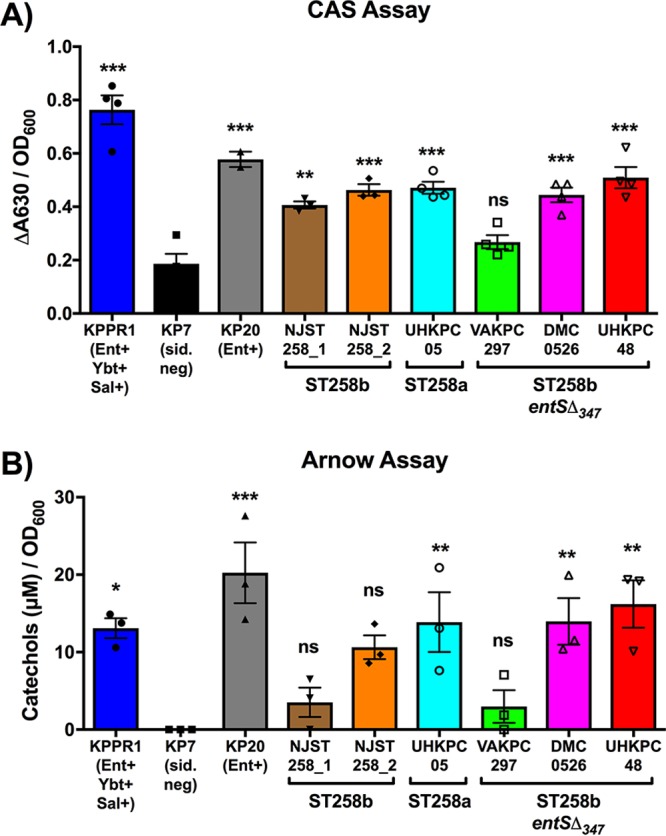
The majority of ST258 strains secrete detectable amounts of iron-chelating molecules and catechols. (A and B) To measure the relative concentration of iron-chelating molecules and catechols secreted by each strain, the CAS and Arnow assays were performed, respectively. Data from filter-sterilized supernatants from overnight cultures in M9 medium normalized for bacterial growth using OD_600_ for each strain from three independent experiments are shown, analyzed using one-way analysis of variance (ANOVA) with Dunnett’s posttest to identify significant differences compared to the siderophore-negative strain, KP7. Values significantly different from the value for strain KP7 are indicated by asterisks as follows: **, *P* < 0.01; ***, *P* < 0.001. Values that are not significantly different (*P* > 0.05) from the value for KP7 are indicated (ns). ΔA630, shift in absorbance at 630 nm.

To explore the ability of ST258 strains of K. pneumoniae to grow in iron-depleted conditions, *in vitro* chrome azurol S (CAS) and Arnow assays examining the secretion of iron-chelating molecules were performed. The CAS assay involves a dye bound to iron that can be removed by a chelator, resulting in a shift in the dye color, and is measured as a shift in absorbance at 630 nm (Δ*A*_630_) (17). To detect iron-chelating molecules present in bacterial culture after overnight growth in M9 medium, supernatants were subjected to the CAS assay. In addition to KPPR1 and KP7 controls, KP20 was used as a control strain that secretes only Ent based on mass spectrometry (18). Supernatants from all strains, except VAKPC297 (*entS*Δ_347_), contained significantly more iron-chelating molecules than KP7, the siderophore negative-control strain, did (Fig. 4A). The lack of secreted iron-chelating molecules by VAKPC297 compared to UHKPC48 is consistent with the growth defect of VAKPC297 in M9/Chelex shown in Fig. 3A. However, the *entS*Δ_347_ strains UHKPC48 and DMC0526 secreted significantly more iron-chelating molecules than strain KP7 did. CAS assay results from strains cultured overnight in M9/Chelex exhibited similar levels of iron-chelating molecules, indicating that residual iron in the M9 minimal medium did not impact the results.

Whereas the CAS assay detects general iron-chelating molecules, the Arnow assay detects catechols, which are the iron coordinating regions of Ent. Similar to the CAS assay, the Arnow assay is a colorimetry-based assay in which a shift in color indicates the presence of catechols (19). To determine whether *entS*Δ_347_ strains secrete catechols, the Arnow assay was performed on bacterium-free culture supernatants. As expected, strains KPPR1 (Ent^+ ^Ybt^+ ^Sal^+^) and KP20 (Ent^+^) secreted significantly more catechols than strain KP7 (siderophore negative) did (Fig. 4B). Strains UHKPC05 (WT *entS*), DMC0526 (*entS*Δ_347_), and UHKPC48 (*entS*Δ_347_) all secreted significant amounts of catechols, and strain NJST258_2 produced a moderate amount (*P* = 0.054). Strain NJST258_1 did not secrete significantly more catechols than KP7 despite secreting significantly more iron-chelating molecules (Fig. 4A). Consistent with CAS assay results, strain VAKPC297 (*entS*Δ_347_) also did not secrete significantly more catechols than KP7 did. Arnow assay results from strains cultured overnight in M9/Chelex exhibited similar results. These results demonstrate that the majority of ST258 strains are secreting detectable levels of iron-chelating molecules and catechols, despite the *entS*Δ_347_ allele in strains DMC0526 and UHKPC48.

### Mass spectrometry detects Ent release by ST258b *entS*Δ_347_ strains.

**FIG 5:**
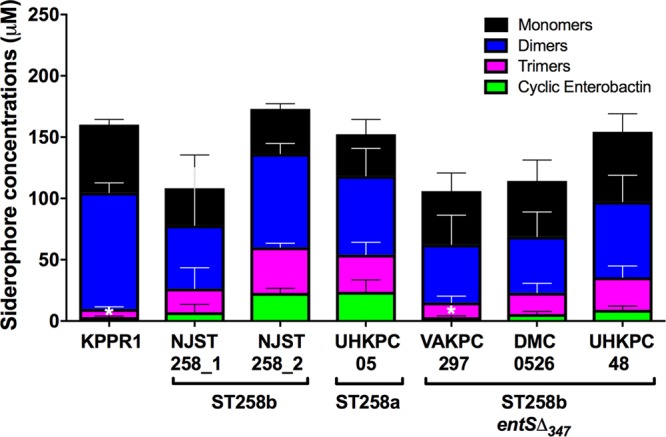
ST258b *entS*Δ_347_ strains secrete Ent. To identify the iron-chelating molecules secreted by ST258 isolates, mass spectrometry was performed. Bacteria were cultured overnight in duplicate in M9/Chelex, and cell-free supernatants were obtained. Concentrations of monomers, dimers, trimers, and cyclic Ent were determined in triplicate and analyzed using one-way ANOVA with Dunnett’s posttest (*, *P* < 0.05 noted for cyclic enterobactin versus NJST258_2. No other comparisons between strains were statistically significant.).

Because the *entS*Δ_347_ genotype did not correlate consistently with phenotype in the CAS and Arnow assays, we used a more definitive method to measure Ent secretion by ST258 strains of K. pneumoniae. Mass spectrometry has been used to detect siderophores in K. pneumoniae, independent of their ability to chelate iron or react with Arnow reagents (18). Mass spectrometry analysis revealed that all ST258 strains of K. pneumoniae secreted monomers, dimers, trimers, and cyclic Ent (Fig. 5), even in the absence of a full-length *entS*. There were no significant differences in the concentrations of monomers, dimers, or overall secretion of Ent products. Compared to the ST258b strain NJST258_2 that contains a full-length copy of *entS*, *entS*Δ_347_ mutant VAKPC297 produced significantly less cyclic Ent. Despite also harboring this *entS*Δ_347_ mutation, strains DMC0526 and UHKPC48 did not produce significantly less cyclic Ent compared to strain NJST258_2. Strain KPPR1 also secreted less cyclic enterobactin, consistent with conversion of a portion of its Ent to Sal (18). These data confirm that ST258 strains secrete Ent despite the *entS*Δ_347_ allele in some ST258b strains.

### CR K. pneumoniae growth in the presence of hemin.

**FIG 6:**
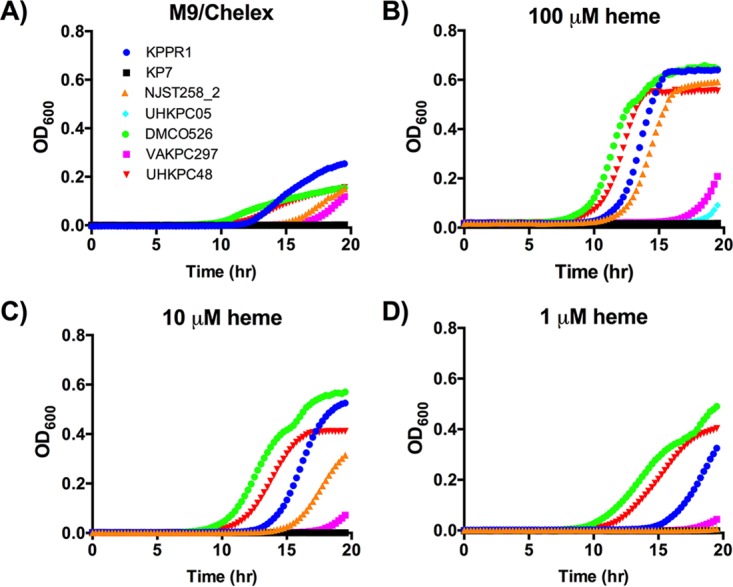
ST258 *entS*Δ_347_ strains can grow iron-depleted media supplemented with heme. (A to D) Growth of strains in M9/Chelex and M9/Chelex supplemented with 100, 10, and 1 µM hemin was determined. Strains were cultured overnight, and OD_600_ was read at 15-min intervals. Data are representative of two individual replicates.

RNA-Seq analysis identified that the *entS*Δ_347_ strains UHKPC48 and VAKPC297 produced increased transcripts for *hmuS*, a hemin transporter, upon growth in M9/Chelex+serum (Fig. 2B). To determine whether *K*. *pneumoniae* strains expressing the *entS*Δ_347_ allele can utilize hemin as an iron source, growth curves were constructed for strains grown in M9/Chelex supplemented with 100, 10, or 1 µM hemin (Fig. 6). As in Fig. 3, KPPR1 and ST258 strains were able to grow in M9/Chelex, whereas KP7 (siderophore-negative) strain could not (Fig. 6A). KPPR1 growth improved with hemin addition, whereas KP7 could not grow, indicating that hemin-dependent growth in this strain required siderophores. Addition of 100, 10, and 1 µM hemin restored growth in two of three *entS*Δ_347_ strains (DMC0526 and UHKPC48), but not in strain VAKPC297 (Fig. 6B to D), indicating that the hemin may be used as an iron source in some *entS*Δ_347_ strains of ST258 K. pneumoniae during growth in iron-depleted conditions.

### Lcn2 inhibits growth of ST258 *entS* mutants.

**FIG 7:**
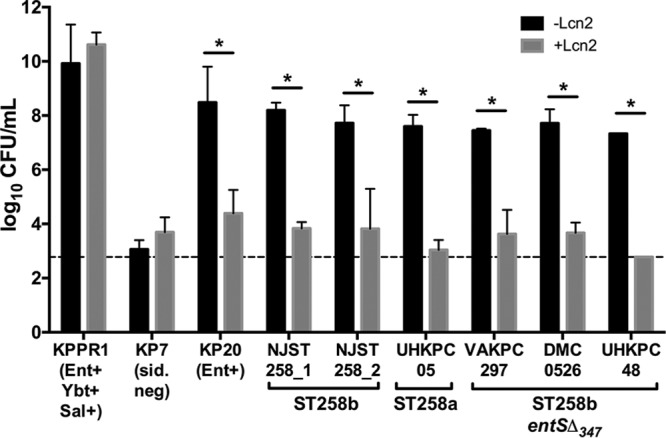
Growth of ST258 strains in serum is inhibited in the presence of Lcn2. Strains were grown in 10% heat-inactivated serum in RPMI 1640 medium with 5 µM Lcn2 (+Lcn2) or without Lcn2 (−Lcn2) overnight and plated for CFU. The dashed line represents the assay limit of detection. Data from two independent experiments were analyzed using one-way ANOVA with Fisher’s posttest. Values that are significantly different (*P* > 0.05) are indicated by a bar and asterisk.

Mass spectrometry analysis identified that strains are releasing Ent, despite a 347-bp deletion in *entS* in a subset of ST258b strains (Fig. 5). This Ent release, whether through an active exporter or another indirect method, could promote growth in iron-limited conditions. Growth in serum by K. pneumoniae requires Ent or Sal due to the presence of transferrin as described above (16). To measure growth in serum that is attributable to Ent, the medium was supplemented with Lcn2, an innate immune protein secreted by host epithelial cells and neutrophils that specifically binds to and sequesters Ent (20). Strain KPPR1 that secretes multiple siderophores is not inhibited by the presence of Lcn2 because it secretes Sal, whereas strain KP20 (Ent^+^) is inhibited by Lcn2 (Fig. 7). The siderophore-negative mutant grows poorly regardless of Lcn2, consistent with media supplemented with 10% serum being a siderophore-dependent growth condition. All ST258 strains tested were able to grow in human serum. However, Lcn2 significantly inhibited growth of all ST258 strains, indicating that *entS*Δ_347_ mutants utilize Ent for serum growth (Fig. 7). Although *entS*Δ_347_ mutants expressed the hemin receptor more than other strains, the ability of strains DMC0526 and UHKPC48 to utilize hemin for growth (Fig. 6) did not prevent serum growth inhibition by Lcn2.

## DISCUSSION

CR K. pneumoniae is resistant to all or nearly all antibiotics and is a major health care concern (1). One CR MLST type in particular, ST258, has disseminated worldwide. Previous studies have identified epidemiological and genomic differences between two prevalent ST258 clades, clade ST258a and ST258b, including a 347-bp deletion (*entS*Δ_347_) in the Ent exporter *entS* gene (8–10). This mutation in a highly conserved iron acquisition system suggested differences in iron metabolism among ST258 strains. To examine the transcriptional response of CR K. pneumoniae to iron, we performed RNA-Seq in iron-depleted and iron-replete conditions to identify transcriptional differences. We identified potential mechanisms of iron acquisition by CR K. pneumoniae: the hemin transport operon that has not been characterized previously in K. pneumoniae, a TonB-dependent siderophore receptor FitA, and a catecholate receptor Fiu. We determined that the hemin transport system is expressed at greater levels in *entS*Δ_347_ strains of ST258b K. pneumoniae compared to strains with intact *entS*. We also characterized the growth patterns and secretion of iron-chelating molecules during *in vitro* iron-limiting conditions. We found that all ST258 isolates, including *entS*Δ_347_ strains, could grow under iron-limiting conditions and secrete iron-chelating molecules, including Ent. Additionally, we found that hemin stimulates growth of two of three ST258b* entS*Δ_347_ strains tested in iron-depleted media. These findings begin to characterize how CR K. pneumoniae bacteria respond to iron limitation.

Transcriptional analysis by RNA-Seq identified genes that were consistently induced across ST258 strains in response to iron limitation (Table 2) and those that were differentially regulated in strains with the *entS*Δ_347_ allele. One gene that was upregulated across ST258 isolates upon iron depletion is *fitA*, a proposed TonB-dependent siderophore receptor (Table 2 and Fig. 1A) (21). In E. coli, the *fit* operon was found to be an iron acquisition mechanism commonly found in human-pathogenic, but not commensal, clinical isolates (21). The substrate for the *fit* operon is unknown; however, Ent is not the substrate (21, 22). The *fit* operon represents a mechanism by which ST258 isolates can acquire iron in an Ent-independent manner. A second gene locus of interest that was upregulated in response to iron-depleted conditions by ST258 isolates is the catecholate receptor Fiu (Table 2 and Fig. 1B). The outer membrane receptor Fiu has been hypothesized to mediate uptake of hydrolytic products of Ent, such as linear 2,3-DHBS monomers (23, 24). It is possible that ST258 isolates, particularly ST258b isolates that are deficient in a full-length *entS*, utilize Fiu to scavenge for and take up hydrolytic products of Ent that may have been secreted through mechanisms other than the dedicated Ent exporter. Indeed, mass spectrometry analysis identified that the majority of Ent products secreted by all strains were hydrolytic products of Ent, rather than cyclic Ent (Fig. 5). There were significant differences between strains in the release of cyclic Ent, but no differences in the concentrations of monomers for isolates. This is consistent with prior work illustrating that *entS* mutants in E. coli secrete 2,3-DHBS (12).

The hemin transport operon was shown to be upregulated by all ST258 isolates in response to iron-depleted conditions. Additionally, it was shown to be significantly upregulated by *entS*Δ_347_ isolates compared to *entS*^*+*^ isolates in M9/Chelex+serum (Fig. 2B). Further *in vitro* characterization of the ST258 isolates determined that strains UHKPC48 and DMC0526 were able to utilize hemin as an iron source upon growth in iron-depleted media, although strain VAKPC297 was not able to utilize hemin for growth (Fig. 6). However, the ability to utilize hemin for growth did not result in growth of the strains in serum upon the addition of Lcn2 (Fig. 7). One possibility is these strains require Ent for hemin utilization. Indeed, strain KPPR1 requires siderophores to utilize hemin for growth *in vitro* (Fig. 6), but this strain has low expression of the hemin receptor in M9 medium (Fig. 2). Alternatively, hemin may be scarce in serum, since it can be scavenged by members of the lipocalin superfamily, in particular α-1-microglobulin (25, 26) and was not sufficient to promote growth via hemin transport in the serum growth assay.

The main limitation of this study was that nonisogenic strains were compared to characterize the phenotype associated with the *entS*Δ_347_ allele. We attempted to mitigate this by focusing on comparisons between clonally related ST258b isolates and performing comparative genomics. Despite containing a 347-bp deletion in *entS*, ST258b isolates UHKPC48 and DMC0526 appear to produce extracellular Ent as measured by the Arnow and CAS assays (Fig. 4). Strain VAKPC297 had detectable Ent, but only when measured by mass spectroscopy. Interestingly, this strain has a nonsynonymous mutation in *entC*, the gene encoding isochorismate synthase in the Ent synthesis pathway, where an alanine has replaced a glycine at position 362 of the protein (GenBank accession no. EOZ46174). If this affects enzymatic function, it may explain lower detected Ent levels. However, all three strains release sufficient Ent to support robust growth in human serum, a matrix relevant to human infection, and that growth is inhibited by Lcn2 (Fig. 7). It is unclear whether Ent supports growth through a specific secretion mechanism or some other form of release. A more complete understanding of iron acquisition by CR K. pneumoniae requires the examination of additional ST258 clinical isolates and development of efficient tools for isogenic mutant construction and ectopic gene expression in these highly antibiotic-resistant strains. In summary, *entS*Δ_347_ strains have altered hemin receptor expression in response to iron limitation, but they retain the ability to release Ent for growth. Although iron acquisition is essential for virulence, this is a new example of the varied strategies used by K. pneumoniae to carry out a vital function.

## MATERIALS AND METHODS

### Bacterial strains and media.

Lab strains of K. pneumoniae, strains KPPR1, KP7 (KPPR1 *entB ybtS*), and KP20 (KPPR1 *iroA ybtS*), have been previously described (27). The clinical isolates NJST258_1 and NJST258_2 (ST258b), provided by Barry Kreiswirth, were previously sequenced (8), while strains UHKPC05 (ST258a), UHKPC48, DMC0526, and VAKPC297 (all ST258b *entS*) were previously sequenced and characterized in references 8 to 10 (see Table S1 in the supplemental material).

10.1128/mSphere.00125-18.4 TABLE S1  Samples and genomes used in RNA-Seq analysis. Download TABLE S1, PDF file, 0.03 MB. Copyright © 2018 Holden et al. 2018 Holden et al. This content is distributed under the terms of the Creative Commons Attribution 4.0 International license.

K. pneumoniae strains were cultured in LB or M9 minimal medium with and without additional supplementation. M9 minimal salts were diluted and prepared according to the manufacturer’s instructions (ThermoFisher, Waltham, MA). To create iron-depleted medium, M9 minimal medium was treated with Chelex 100 resin for 1 h at room temperature (Bio-Rad). Following Chelex treatment, M9 was supplemented with d-glucose, MgSO_4_, and CaCl_2_ to generate M9/Chelex. M9/Chelex was supplemented with 100 µM Fe_2_SO_4_ (M9/Chelex+Fe), 5% heat-inactivated (HI) human serum (M9/Chelex+serum) (Sigma, St. Louis, MO), or 1, 10, or 100 µM hemin (M9/Chelex + hemin) (Sigma) where indicated.

### Growth curves.

Growth curves were performed as previously described (27).

### RNA-Seq experiments.

Representative strains were cultured in duplicate overnight in LB broth and subcultured 1:100 into fresh LB until bacterial growth reached mid-log phase. At an optical density at 600 nm (OD_600_) of ~0.3, bacteria were plated for CFU and subcultured 1:10 from the mid-log LB culture into M9/Chelex, M9/Chelex+Fe, or M9/Chelex+serum in duplicate. After 1-h incubation, the OD_600_ for each sample was read, samples were plated for CFU (Fig. S2), and two aliquots of 10^8^ bacteria were preserved in 2× volumes of RNAprotect bacterial reagent (Qiagen, Germantown, MD). Samples were frozen at −80°C until RNA was isolated.

Thawed samples were resuspended with 500 µl ice-cold sterile Dulbecco’s phosphate-buffered saline (DPBS) to remove RNAprotect bacterial reagent. Samples were spun for 10 min at 10,000 rpm to repellet bacteria. RNA was isolated from 10^8^ CFU of bacteria using the MagJET RNA purification kit (ThermoFisher) and then treated with an additional DNase treatment. rRNA was depleted using Ribo-Zero (Illumina, San Diego, CA). A directional transcriptome sequencing (RNA-Seq) library was constructed using PrepX RNA-Seq for Illumina library kit (Wafergen, Fremont, CA), which was then quantified and normalized using quantitative PCR (qPCR). Samples were sequenced using Illumina NextSeq 500 mid output kit (75-bp paired end), and paired-end reads were mapped to each respective genome using CLC Genomics Workbench (Qiagen). For each gene, raw counts and transcripts per million (TPM) values were calculated. Orthologs in each genome were identified using PanOCT to enable gene expression comparisons across genomes (28). Differential expression analysis was conducted using the DESeq2 package in R.

### CAS assay.

The chrome azurol S (CAS) assay was performed as previously described to determine the iron-chelating capabilities of bacterial supernatants (29). Briefly, bacteria were inoculated into M9 medium overnight. OD_600_ readings were taken, and bacterial cultures were spun through a 0.2-µm filter to remove bacteria. Supernatants were subjected to the CAS assay, and the change in absorbance at 630 nm was read. Results are expressed in Δ*A*_630_/OD_600_ for each culture to normalize for differences in bacterial growth.

### Arnow assay.

The Arnow assay was performed as previously described to determine the secretion of catechols in bacterial cultures (30). Briefly, strains were cultured overnight in M9 medium, OD_600_ was read, and bacterium-free supernatants were acquired using 0.2-µm filters. Catechols were measured by creating an equal mix of sample, 0.5 N HCl, nitrite-molybdate reagent (10% [wt/vol] sodium nitrate and sodium molybdate), and 1 N NaOH. Absorbance was read at 510 nm, and samples were normalized for bacterial growth using the OD_600_ of each culture.

### Lcn2 growth assay.

The Lcn2 growth assay was performed as previously described (18) to measure lipocalin-mediated iron limitation.

### Mass spectrometry.

Strains were grown overnight in duplicate in M9/Chelex and centrifuged to pellet bacteria. Supernatants were passed through a 0.2-µm-pore-size filter to remove remaining bacteria and frozen at −80°C until analysis. Siderophore concentrations were determined by mass spectrometry as previously described (18, 31, 32).

### Data analysis.

Statistical analysis for *in vitro* assays was performed using GraphPad Prism.

### Accession number(s).

RNA-Seq data have been submitted to the NCBI Sequence Read Archive under the following accession numbers: SAMN04330238, SAMN04330239, SAMN04330240, SAMN04330241, SAMN04330242, SAMN04330243, SAMN04330244, SAMN04330245, SAMN04330246, SAMN04330247, SAMN04330248, SAMN04330249, SAMN04330250, SAMN04330251, SAMN04330252, SAMN04330253, SAMN04330254, SAMN04330232, SAMN04330233, SAMN04330234, SAMN04330235, SAMN04330236, SAMN04330237, SAMN04330226, SAMN04330227, SAMN04330228, SAMN04330229, SAMN04330230, and SAMN04330231. See Table S1 for details.
