# Supplementary material for: Iron Acquisition and Siderophore Release by Carbapenem-Resistant Sequence Type 258 Klebsiella pneumoniae
Source: mSphere. 2018 Apr 18;3(2):e00125-18. doi: 10.1128/mSphere.00125-18 (PMC5907654; doi:10.1128/mSphere.00125-18)
Supplement: FIG S1 [file sph002182514sf1.pdf]

A)

```
ST258B          ATGAACCGACAATCCTGGCTGCTCAATCTCAGCCTGCTGAAAACTCACCCGGCGTTTCGC
ST258A          ATGAACCGACAATCCTGGCTGCTCAATCTCAGCCTGCTGAAAACTCACCCGGCGTTTCGC
                ************************************************************

ST258B          GCCGTTTTTATCGCTCGCTTTATCTCTATTCTGTCGCTTGGCCTGCTGGGCGTGGCCATT
ST258A          GCCGTTTTTATCGCTCGCTTTATCTCTATTCTGTCGCTTGGCCTGCTGGGCGTGGCCATT
                ************************************************************

ST258B          CCGGTGCAGATCCAGATGATGACCCATTCGACCTGGCAGGTCGGGCTGTCGGTGACGCTG
ST258A          CCGGTGCAGATCCAGATGATGACCCATTCGACCTGGCAGGTCGGGCTGTCGGTGACGCTG
                ************************************************************

ST258B          ACCGGGGCGTCGATGTTTGTTGGTCTGATGGTGGGGGGCGTGCTGGCTGACCGCTATGAA
ST258A          ACCGGGGCGTCGATGTTTGTTGGTCTGATGGTGGGGGGCGTGCTGGCTGACCGCTATGAA
                ************************************************************

ST258B          CGTAAACGCCTGATCCTGCTGGCGCGCGGCACCTGCGGCGTGGGCTTTGTCGGCCTGTGT
ST258A          CGTAAACGCCTGATCCTGCTGGCGCGCGGCACCTGCGGCGTGGGCTTTGTCGGCCTGTGT
                ************************************************************

ST258B          CTGAACGCCCTGCTGCCGGAGCC-------------------------------------
ST258A          CTGAACGCCCTGCTGCCGGAGCCGTCGCTGGCGGCGATCTACCTGCTGGGGATCTGGGAC
                ***********************

ST258B          ------------------------------------------------------------
ST258A          GGGTTCTTCGCCTCGTTGGGGGTGACCGCGCTGCTGGCGGCGACCCCGGCGCTGGTGGGA

ST258B          ------------------------------------------------------------
ST258A          CGGGAAAACCTGATGCAGGCCGGGGCCATCACTATGTTGACGGTGCGCCTGGGCTCGGTG

ST258B          ------------------------------------------------------------
ST258A          ATTTCGCCGATGATCGGCGGCCTGCTGCTGGCCACCGGCGGCGTGGCCTGGAACTTTGGC

ST258B          ------------------------------------------------------------
ST258A          CTGGCGGCGGCGGGGACCTTTATCACCACCTTAACCCTGCTGCGTCTGCCGCAGCTGCCG

ST258B          ------------------------------------------------------------
ST258A          CCGCCTCCGCAGCCGCGCGAGCATCCGCTGCGCTCCCTGCTGGCGGGGCTGACCTTCCTC

ST258B          ----------CGCTTATCGGCGGGATTGCGCTGCTTGGCGGTCTGCTGACCATGGCCAGC
ST258A          TGCCGGAGCCCGCTTATCGGCGGGATTGCGCTGCTTGGCGGTCTGCTGACCATGGCCAGC
                          **************************************************

ST258B          GCGGTGCGGGTGCTCTATCCGGCGCTGGCCGGTAGCTGGCAGATGTCGGCCGGACAGATT
ST258A          GCGGTGCGGGTGCTCTATCCGGCGCTGGCCGGTAGCTGGCAGATGTCGGCCGGACAGATT
                ************************************************************

ST258B          GGCCTGCTGTACGCCGCTATTCCGCTCGGTGCGGCGCTGGGGGCGTTGACCAGCGGCCAG
ST258A          GGCCTGCTGTACGCCGCTATTCCGCTCGGTGCGGCGCTGGGGGCGTTGACCAGCGGCCAG
                ************************************************************

ST258B          CTGGCCCAGACGGTGCGGCCGGGCGCGCTGATGCTGGCGACGACGGTGGGATCGTTTGTC
ST258A          CTGGCCCAGACGGTGCGGCCGGGCGCGCTGATGCTGGCGACGACGGTGGGATCGTTTGTC
                ************************************************************

ST258B          GCCATTGCGCTGTTCAGCCTGATGCCGCACTGGGCATTGGGCGCGCTGTGCCTGGCGCTG
ST258A          GCCATTGCGCTGTTCAGCCTGATGCCGCACTGGGCATTGGGCGCGCTGTGCCTGGCGCTG
                ************************************************************

ST258B          TTTGGCTGGCTGAGCGCTATTAGCTCGCTGCTGCAGTACACCCTGATTCAGACCCAGACG
ST258A          TTTGGCTGGCTGAGCGCTATTAGCTCGCTGCTGCAGTACACCCTGATTCAGACCCAGACG
                ************************************************************
```

```
ST258B          CCGGAACATATGCTCGGGCGGATTAACGGTCTGTGGACCGCGCAAAACGTCACCGGCGAC
ST258A          CCGGAACATATGCTCGGGCGGATTAACGGTCTGTGGACCGCGCAAAACGTCACCGGCGAC
                ************************************************************

ST258B          GCCATCGGCGCGGCGCTGCTCGGCGGCTTAGGGGCGGTAATGACGCCGGCGGCATCCGCC
ST258A          GCCATCGGCGCGGCGCTGCTCGGCGGCTTAGGGGCGGTAATGACGCCGGCGGCATCCGCC
                ************************************************************

ST258B          AGCGCCAGCGGCTGGGCGCTGGCGCTTGTCGGTGTGCTGCTGGTTGGGCTGCTACGCGAG
ST258A          AGCGCCAGCGGCTGGGCGCTGGCGCTTGTCGGTGTGCTGCTGGTTGGGCTGCTACGCGAG
                ************************************************************

ST258B          CTGCGCCGTTTCCAGCGCCCGGAAATTGTCAACGAAAGTTAA
ST258A          CTGCGCCGTTTCCAGCGCCCGGAAATTGTCAACGAAAGTTAA
                ******************************************
```

B)

```
ST258a      1 MNRQSWLLNLSLLKTHPAFRAVFIARFISILSLGLLGVAIPVQIQMMTHSTWQVGLSVTL
ST258b      1 MNRQSWLLNLSLLKTHPAFRAVFIARFISILSLGLLGVAIPVQIQMMTHSTWQVGLSVTL
              ************************************************************

ST258a     61 TGASMFVGLMVGGVLADRYERKRLILLARGTCGVGFVGLCLNALLPEPSLAAIYLLGIWD
ST258b     61 TGASMFVGLMVGGVLADRYERKRLILLARGTCGVGFVGLCLNALLPEPAYRRDCAAWRSA
              ************************************************

ST258b    121 GFFASLGVTALLAATPALVGRENLMQAGAITMLTVRLGSVISPMIGGLLLATGGVAWNFG
ST258b    121 DHGQRGAGALSGAGR---------------------------------------------

ST258a    181 LAAAGTFITTLTLLRLPQLPPPPQPREHPLRSLLAGLTFLCRSPLIGGIALLGGLLTMAS
ST258b    181 ------------------------------------------------------------

ST258a    241 AVRVLYPALAGSWQMSAGQIGLLYAAIPLGAALGALTSGQLAQTVRPGALMLATTVGSFV
ST258b    241 ------------------------------------------------------------

ST258a    301 AIALFSLMPHWALGALCLALFGWLSAISSLLQYTLIQTQTPEHMLGRINGLWTAQNVTGD
ST258b    301 ------------------------------------------------------------

ST258a    361 AIGAALLGGLGAVMTPAASASASGWALALVGVLLVGLLRELRRFQRPEIVNES
ST258b    361 -----------------------------------------------------
```

Figure S1
