# Supplementary figures and images for: Iron Acquisition and Siderophore Release by Carbapenem-Resistant Sequence Type 258 Klebsiella pneumoniae
Source: mSphere. 2018 Apr 18;3(2):e00125-18. doi: 10.1128/mSphere.00125-18 (PMC5907654; doi:10.1128/mSphere.00125-18)

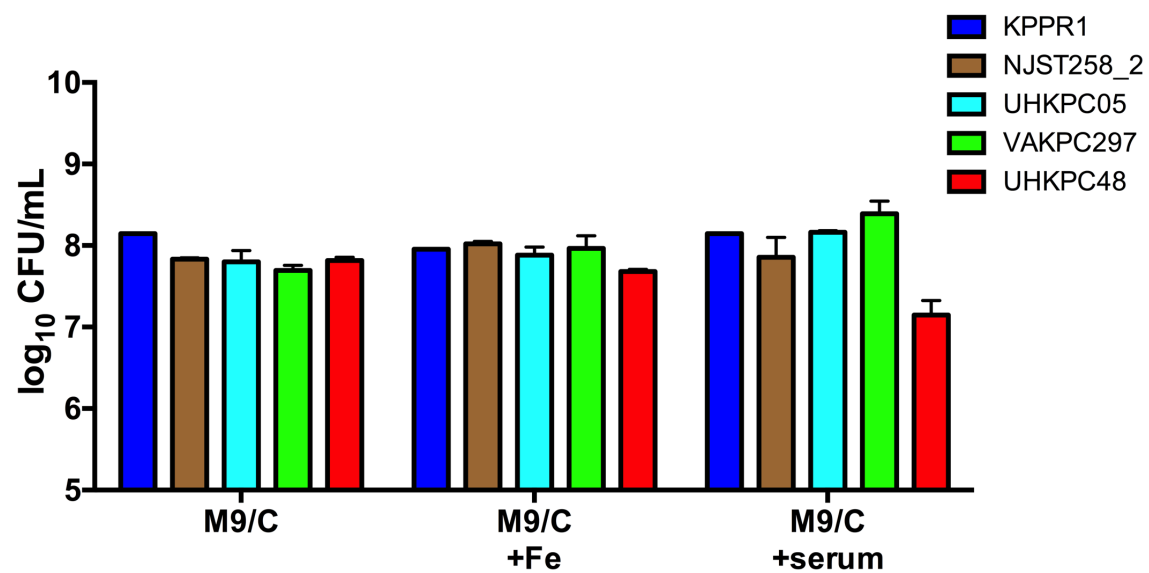

Figure S2

Supplement: FIG S2 [file sph002182514sf2.pdf]

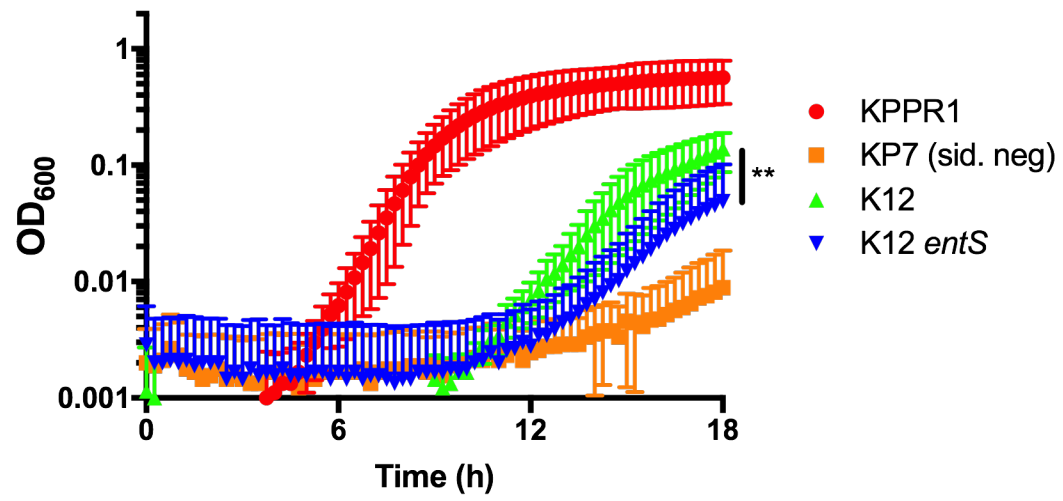

Figure S3

Supplement: FIG S3 [file sph002182514sf3.pdf]
