# Supplementary material for: Iron Acquisition and Siderophore Release by Carbapenem-Resistant Sequence Type 258 Klebsiella pneumoniae
Source: mSphere. 2018 Apr 18;3(2):e00125-18. doi: 10.1128/mSphere.00125-18 (PMC5907654; doi:10.1128/mSphere.00125-18)
Supplement: TABLE S1 [file sph002182514st1.pdf]

Table S1

BioSample_s	Experiment_s	Sample_Name_s	Run_s	SRA_Sample_s	MBases_l	WGS RefSeq Accession	WGS Biosample
SAMN04330238	SRX1485614	UHKPC05M9 _Rep_1	SRR3018848	SRS1208831	646	GCF_000709245	SAMN02141987
SAMN04330239	SRX1485615	UHKPC05M9 _Rep_2	SRR3018849	SRS1208834	682		
SAMN04330240	SRX1485616	UHKPC05M9FE _Rep_1	SRR3018850	SRS1208833	563		
SAMN04330241	SRX1485617	UHKPC05M9FE _Rep_2	SRR3018851	SRS1208832	619		
SAMN04330242	SRX1485620	UHKPC05M9SERUM _Rep_1	SRR3018857	SRS1208837	589		
SAMN04330243	SRX1485621	UHKPC05M9SERUM _Rep_2	SRR3018858	SRS1208836	623		
SAMN04330244	SRX1485622	UHKPC48M9 _Rep_1	SRR3018859	SRS1208838	817	GCF_000409085	SAMN02142018
SAMN04330245	SRX1485623	UHKPC48M9 _Rep_2	SRR3018861	SRS1208840	843		
SAMN04330246	SRX1485628	UHKPC48M9FE _Rep_1	SRR3018863	SRS1208843	767		
SAMN04330247	SRX1485637	UHKPC48M9FE _Rep_2	SRR3018907	SRS1208852	736		
SAMN04330248	SRX1485691	UHKPC48M9SERUM _Rep_1	SRR3019181	SRS1208860	622		
SAMN04330249	SRX1485692	UHKPC48M9SERUM _Rep_2	SRR3019182	SRS1208859	593		
SAMN04330250	SRX1485693	VAKPC297M9 _Rep_1	SRR3019183	SRS1208858	669	GCF_000406545	SAMN02142050
SAMN04330251	SRX1485694	VAKPC297M9 _Rep_2	SRR3019185	SRS1208861	607		
SAMN04330252	SRX1485711	VAKPC297M9FE _Rep_1	SRR3019202	SRS1208862	619		
SAMN04330253	SRX1485712	VAKPC297M9SERUM _Rep_1	SRR3019203	SRS1208879	820		
SAMN04330254	SRX1485713	VAKPC297M9SERUM _Rep_2	SRR3019204	SRS1208880	687		
SAMN04330232	SRX1485608	NJST2582M9 _Rep_1	SRR3018841	SRS1208826	698	GCF_000597905	SAMN03081501
SAMN04330233	SRX1485609	NJST2582M9 _Rep_2	SRR3018842	SRS1208825	639		
SAMN04330234	SRX1485610	NJST258M9FE _Rep_1	SRR3018843	SRS1208827	733		
SAMN04330235	SRX1485611	NJST258M9FE _Rep_2	SRR3018844	SRS1208829	633		
SAMN04330236	SRX1485612	NJST258M9SERUM _Rep_1	SRR3018845	SRS1208828	751		
SAMN04330237	SRX1485613	NJST258M9SERUM _Rep_2	SRR3018846	SRS1208830	642		
SAMN04330226	SRX1485596	KP4M9 _Rep_1	SRR3018830	SRS1208818	555	GCF_000742755	SAMN02982872
SAMN04330227	SRX1485598	KP4M9 _Rep_2	SRR3018831	SRS1208816	755		
SAMN04330228	SRX1485599	KP4M9FE _Rep_1	SRR3018832	SRS1208815	673		
SAMN04330229	SRX1485602	KP4M9FE _Rep_2	SRR3018836	SRS1208820	535		
SAMN04330230	SRX1485603	KP4M9SERUM _Rep_1	SRR3018837	SRS1208823	694		
SAMN04330231	SRX1485604	KP4M9SERUM _Rep_2	SRR3018838	SRS1208822	672		
